# FunMappOne: a tool to hierarchically organize and visually navigate functional gene annotations in multiple experiments

**DOI:** 10.1186/s12859-019-2639-2

**Published:** 2019-02-15

**Authors:** Giovanni Scala, Angela Serra, Veer Singh Marwah, Laura Aliisa Saarimäki, Dario Greco

**Affiliations:** 1 Faculty of Medicine and Life Sciences, University of Tampere, Arvo Ylpön katu 34 - Arvo building, Tampere, FI-33014 Finland; 2 BioMediTech Institute, University of Tampere, Arvo Ylpön katu 34 - Arvo building, Tampere, FI-33014 Finland; 3 Institute of Biotechnology, University of Helsinki, Viikinkaari 5d, Helsinki, FI-00014 Finland

**Keywords:** Functional annotation, Pathway visualization, Ontology visualization, KEGG, Gene Ontology, Reactome, R-Shiny

## Abstract

**Background:**

Functional annotation of genes is an essential step in omics data analysis. Multiple databases and methods are currently available to summarize the functions of sets of genes into higher level representations, such as ontologies and molecular pathways. Annotating results from omics experiments into functional categories is essential not only to understand the underlying regulatory dynamics but also to compare multiple experimental conditions at a higher level of abstraction. Several tools are already available to the community to represent and compare functional profiles of omics experiments. However, when the number of experiments and/or enriched functional terms is high, it becomes difficult to interpret the results even when graphically represented. Therefore, there is currently a need for interactive and user-friendly tools to graphically navigate and further summarize annotations in order to facilitate results interpretation also when the dimensionality is high.

**Results:**

We developed an approach that exploits the intrinsic hierarchical structure of several functional annotations to summarize the results obtained through enrichment analyses to higher levels of interpretation and to map gene related information at each summarized level. We built a user-friendly graphical interface that allows to visualize the functional annotations of one or multiple experiments at once. The tool is implemented as a R-Shiny application called FunMappOne and is available at https://github.com/grecolab/FunMappOne.

**Conclusion:**

FunMappOne is a R-shiny graphical tool that takes in input multiple lists of human or mouse genes, optionally along with their related modification magnitudes, computes the enriched annotations from Gene Ontology, Kyoto Encyclopedia of Genes and Genomes, or Reactome databases, and reports interactive maps of functional terms and pathways organized in rational groups. FunMappOne allows a fast and convenient comparison of multiple experiments and an easy way to interpret results.

**Electronic supplementary material:**

The online version of this article (10.1186/s12859-019-2639-2) contains supplementary material, which is available to authorized users.

## Background

Functional annotation of large sets of significant genes is often the final step of omics data analysis. However, when multiple genes are selected during differential analysis, it becomes almost impossible to understand the altered biological processes by manually inspecting the individual genes. This task is even more difficult when comparing functional profiles derived from two or more related experiments at the gene level, for different sets of functionally related genes may be specifically affected in different experimental conditions.

A multitude of tools are already available to the community to graphically represent enriched functional annotations from single pair-wise comparisons [1–4]. When considering multiple experiments, these methods require to run separate analyses for each experiment and subsequently collate the results for comparison. The complexity of this task increases with the number of considered experiments, especially for users who are not familiar with advanced techniques of data manipulation. Some tools allow the visualization of the enriched Gene Ontology terms from multiple experiments [5–7]. However, as they are typically implemented in R, they require a certain degree of programming expertise in order to produce the desired visualizations. Moreover, since these methods usually offer a static graphical output, the produced plots become difficult to read and interpret when large number of functional terms need to be displayed.

An important aspect of some functional annotations is the possibility to derive a hierarchical structure for their base terms, such as for Kyoto Encyclopedia of Genes and Genomes (KEGG) pathways [8], Reactome pathways [9] and Gene Ontology terms [10]. This structure can be used to organize the functional terms and summarize sets of related functions in super classes. This feature can be further exploited to reduce the dimensionality of sets of enriched terms and to abstract the underlying biological functions to higher levels of interpretation.

Here we present FunMappOne, an R-shiny user-friendly software with a simple graphical interface that takes in input lists of human or mouse genes from multiple experiments, optionally with their gene-associated metrics, such as fold change and *p*-value. It provides functionalities to statistically evaluate over-represented biological terms from Gene Ontology, KEGG, or Reactome databases, graphically summarize, and navigate them.

## Method

### The three-level hierarchy

In order to reduce the dimensionality of the sets of enriched terms, we introduced the concept of hierarchical summarization, that is the possibility to explore enriched terms at higher functional levels. To do this, a hierarchy is needed to group terms in super-classes. By definition, this structure needs to be represented as a direct acyclic graph, with a root category (representing the functional annotation) and a series of meta-terms (real terms or functional groups), defining progressively specialized group of terms. This structure is naturally found in the intrinsic organization of KEGG and Reactome pathways while it can be easily derived for Gene Ontology terms, as described in the next section. An important factor for the hierarchy definition and construction is the number of levels of the hierarchy, namely the depth of the corresponding graph structure: KEGG has an intrinsic structure based on three levels, while Reactome pathways and gene ontology can have more than three levels that are not uniformly distributed (the hierarchical chain of meta-terms can have different length for different terms). Having many summarization levels has the advantage of making more specialized grouping of terms but would also complicate the task for the user to reduce the set dimensionality and obtain easier views of the enrichment data. For this reason, we chose to follow the KEGG philosophy and homogenize the three hierarchies (KEGG, Reactome and Gene Ontology) in order to have three levels of summarization from the terms to the root. The detailed implementation of the hierarchies is described in the following section.

### Hierarchy definition

**Fig. 1:**
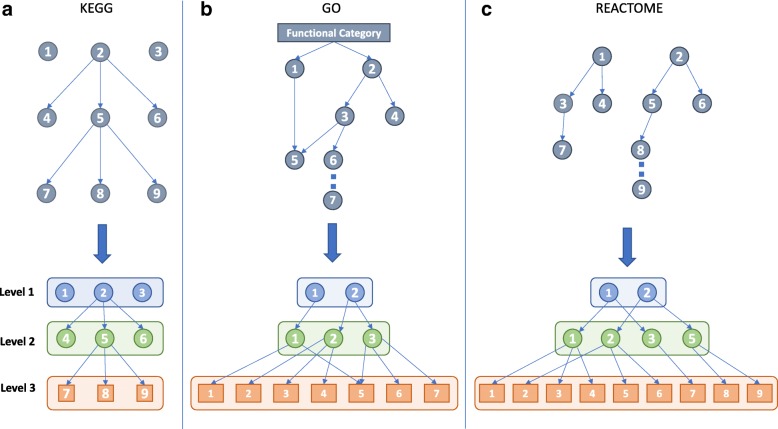
Definition of the hierarchies. For each functional annotation type, a model reflecting the relationship between functional terms and levels in their original structure is shown above the corresponding generated hierarchy. Panel **a**, **b** and **c** report hierarchy generation models for KEGG, Gene Ontology and Reactome, respectively. In the second and third panel, different numbers indicate different functional terms. In panel **b**, “CAT” can be one of the Gene Ontology categories BP, CC or MF
- For each Gene Ontology category CAT (Biological Processes - BP, Cellular Components - CC, and Molecular Functions - MF), a three-level hierarchy was extracted by first considering the graph GO_CAT rooted in CAT (Fig. 1b). Then, the acyclic directed subgraph GO_CAT_ac was computed by considering only the edges representing the relationship “is_a” or “part_of” in GO_CAT. Finally, a new graph GO_CAT_hier was built by considering all the nodes in GO_CAT_ac, and adding, for each node *t*_*i*_, all the edges in the path [*t*_*i*_,…,*t*_*r*−1_] if the path [ *t*_*i*_,…,*t*_*r*−1_,*CAT*] of length at most 3 already existed in GO_CAT_ac. For the paths [ *t*_*i*_,…,*t*_*r*−2_,*t*_*r*−1_,*CAT*] in GO_CAT_ac of length greater than 3, only the arcs forming the sequence [ *t*_*i*_,*t*_*r*−2_,*t*_*r*−1_] were added to GO_CAT_hier.
- For the Reactome pathways (Fig. 1C), the set of root nodes Rs were considered and a three-level hierarchy was explicated. First, the associated graph *REACT*_*RS*_*i*_ rooted in CAT was selected. Next, for each node *t*_*i*_ the edges [*t*_*i*_,*t*_*r*−1_,*RS*_*i*_] were added if the path [*t*_*i*_,*t*_*r*−1_,*RS*_*i*_] belonged to *REACT*_*RS*_*i*_. If the path [*t*_*i*_,…,*t*_*r*−2_,*t*_*r*−1_,*RS*_*i*_] existed in *REACT*_*RS*_*i*_, only the edges forming the sequence [*t*_*i*_,*t*_*r*−2_,*RS*_*i*_] were added to the new graph representing the hierarchy.

Figure 1 shows the implemented procedure to define hierarchical structures for KEGG pathways (panel A), Gene Ontology terms (panel B) and Reactome pathways (panel C), respectively. For each annotation type, a three-level hierarchy was defined.

- For KEGG pathways (Fig. 1a), the three levels of BRITE functional hierarchy was used [8].

### FunMappOne algorithm workflow

**Fig. 2:**
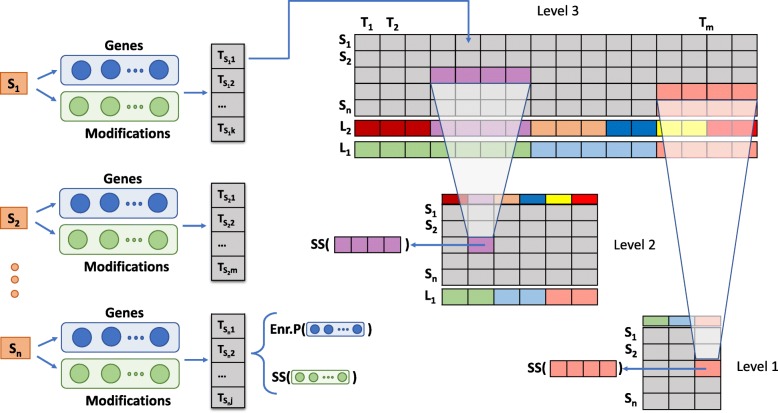
FunMappOne workflow. The tool accepts as input gene lists and modification values for every experimental condition *S*_1_,…,*S*_*n*_ for which the enrichment will be carried out. The analysis performed on the j-th sample will results in a set of enriched terms *T*_*sj*1_,…,*T*_*sjk*_ with an associated *p*-value (Enr.P) from the enrichment function applied on the gene list, or a value coming from the application of a summary statistic (SS) on the associated modification values. A matrix with *n* rows associated to samples and *m* columns associated with the enriched terms is then specified to represent the data structure beneath Level 3 representation of the data. Matrices associated to higher hierarchical levels are composed by *n* rows and as many columns as the categories of the level. Each cell of a higher level matrix contains a value obtained by applying SS to the terms belonging to the associated category from the Level 3 matrix

Figure 2 shows the FunMappOne algorithm workflow. The input is provided as N lists of genes, one for each experimental condition to compare and, optionally, N lists of modifications (e.g. the fold-change or the *p*-value) associated with each gene. For each experiment analyzed, the enriched terms in the chosen functional annotation are computed by using the gProfiler R package [4], and a matrix Ter[NxM] is created, where M is the total number of enriched terms. Each element Ter[i,j] is associated with the hypergeometric test *p*-value of term j for the genes in the i-th list. Optionally, Ter[i,j] can also be associated with a value that summarizes the modification values (e.g. the median fold change) of the genes from the i-th list intersecting the gene set of the term j.

To summarize the information at a higher level of interpretation, a new matrix *Ter*_*i*_[NxK] is created, where i =1,2 is the desired height of the chosen annotation hierarchy and K is the number of different terms at level i. Each element *Ter*_*i*_[i,j] is then associated with a summary statics (e.g. the median *p*-value) of the elements Ter[i,k] for all k such that the term k is a descendant j in the reference hierarchy.

Finally, given a matrix *Ter*_*i*_[NxK] representing the enrichment at level *i* as defined above, the possibility to reorder and cluster experiments, based on a given distance function *D*_*k*,*l*_, is implemented. This is computed between the vectors *Ter*_*i*_[k,] and *Ter*_*i*_[l,] using, alternatively, a distance based on the Jaccard index on the number of common enriched terms, the Euclidean distance on the values associated with terms, or a combination of these two.

In the first case, the Jaccard index *J*_*k*,*l*_ is computed as $\frac {|Terms(k) \cap Terms(l)|}{|Terms(k) \cup Terms(l)|}$, where *Terms*(*x*) is the set of enriched terms for the experiment *x* and *D*_*k*,*l*_ is set as 1−*J*_*k*,*l*_.

In the second case, the set *comm*(*k*,*l*)=*Terms*(*k*)∩*Terms*(*l*)| is first considered, where *Terms*(*x*) is the set of enriched terms for the experimental condition *x*, then if |*comm*(*k*,*l*)|≥0 the Euclidean distance *DE*_*k*,*l*_ on the sub-vectors *Ter*_*i*_[*k*,*comm*] and *Ter*_*i*_[*l*,*comm*] is computed. A combination of the two methods is implemented by creating the mean distance matrix *M*_*k*_=(*D*+*DE*_01_)/2, where *D* is the matrix of the Jaccard index and *DE*_01_ is the Euclidean distance matrix scaled in the range [0,1]. In this way, the experimental conditions are clustered together not only when they share the same enriched terms, but also considering how similar are the enriched terms with respect to their enrichment *p*-value or summary statistic. A hierarchical clustering function is then applied to the matrix using a linkage method between complete, single and ward.

## Results and discussion

The analytical approach presented above was implemented using R-shiny. The typical analysis is performed by three interaction steps: i) input of gene lists and modifications, ii) graphical visualization of enriched terms and iii) interactive navigation of the results. A step-by-step user manual is available in Additional file 1.

In the first step, the application provides a simple graphical interface, where the user can submit a spreadsheet file with the lists of genes associated to each experimental condition of interest and (optionally) their modification information (e.g. the associated fold change from a differential expression analysis). The input spreadsheet contains a sheet for every experimental condition, named with a condition id. In every sheet, two columns are provided, containing the gene identifiers (Entrez Gene, Gene Symbol, or Ensembl gene ids) and, optionally, their modifications, respectively. Furthermore, an additional sheet is required, containing two columns with the condition id and the condition grouping information, respectively.

The user is then asked to choose the species (human or mouse), a functional annotation (Gene Ontology - BP, Gene Ontology - CC, Gene Ontology - MF, KEGG, Reactome), a summarization function (min, median, mean, max) to annotate and summarize the enriched terms with provided modifications, a *p*-value correction method (gSCS [4], bonferroni, fdr), and a statistical significance threshold for the enriched functional terms. If the amplitude of gene modification (e.g. fold change, *p*-value) is provided, the user selects whether the summarized value of the enriched terms is plotted in a color-scale associated to its value, or with three colors only (negative, zero, positive); this latter feature is useful when emphasis is given to the dominant sign of the modification in the term. Moreover, if gene modification values are provided in the input, the user can choose the type of information that will be associated to the enriched terms: the term enrichment *p*-value, the provided modification value, or a combination of term enrichment *p*-values (Enr.P) and modification values (MVs), specified as *MV*×−*log*(*Enr*.*P*). Alternatively, if only gene lists without providing modification values are uploaded, the enrichment *p*-value for each enriched term will be displayed.

After loading the needed files, a dedicated panel in the software graphical environment shows the content of the provided tables, along with a summary of each column.

**Fig. 3:**
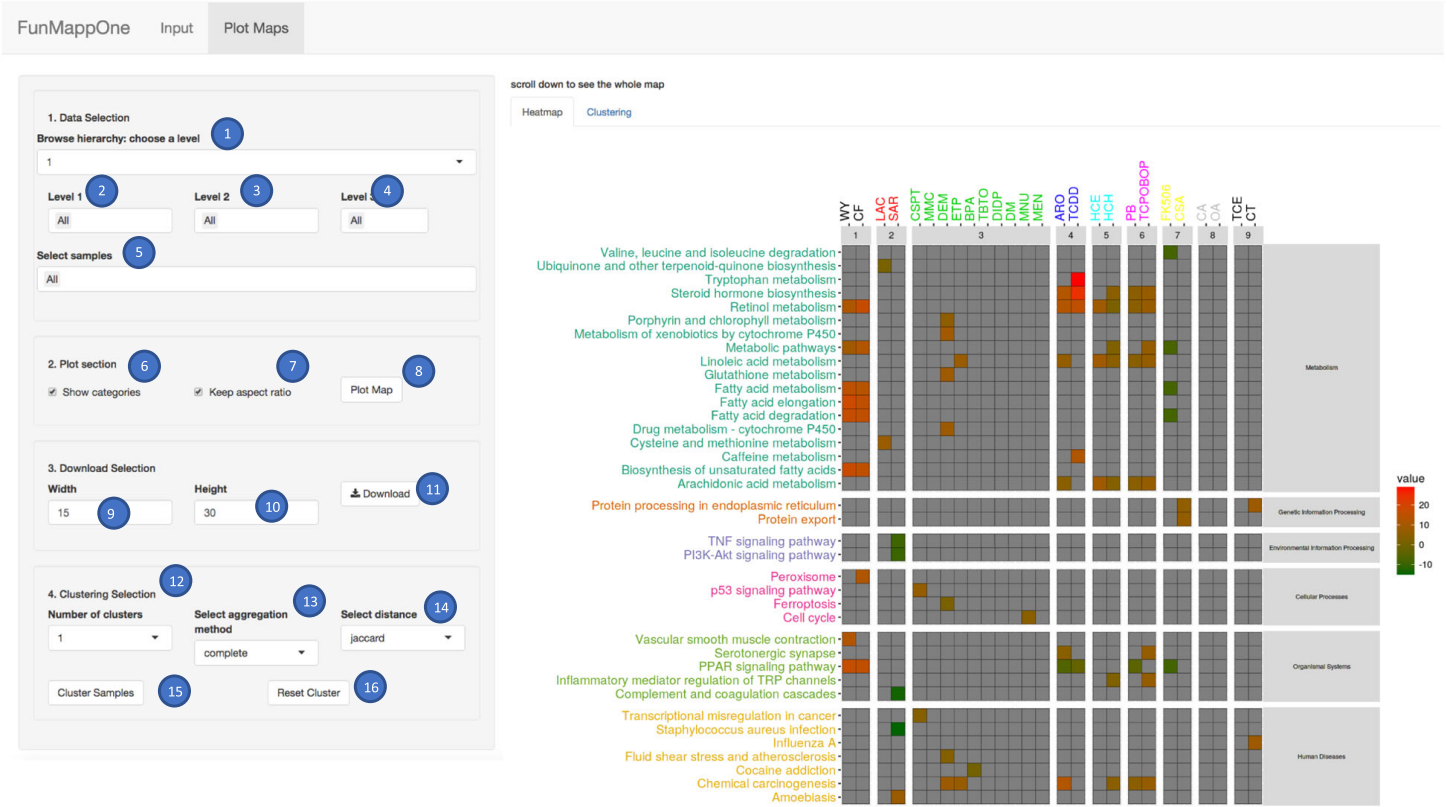
Interactive Map Visualization. The user can select the level of hierarchy to visualize (1) as well as a subset of elements to be plotted at each level of hierarchy (2-4). Furthermore, the user can select a subset of the conditions (5). In the “Plot section” the user can select to show the categories (6) and to keep the aspect ratio (7) for the plot. By clicking the button “Plot Map” (8) the updated map is visualized. After specifying the desired height (9) and width (10) for the pdf that will be downloaded, the user can save the image by clicking the “Download” button (11). Experiments can be clustered by selecting the number of clusters (12), the desired clustering function (13), the distance function (14), and then clicking the “Cluster samples” button (15). The map can be reset to the initial visualization with the predefined grouping by clicking the “Reset cluster” button (16)

After clicking the “Generate Map” button, the tool computes the enrichment and shows the “Plot Maps” panel. After selecting the desired visualization options and clicking the “Plot Map” button, the tool shows the map of enriched terms as a grid (Fig. 3), where columns represent experimental conditions, eventually grouped based on the provided information, and rows represent the enriched terms grouped and colored based on the corresponding hierarchy class.

The user can interact with the generated enrichment map in three different ways: i) by selecting the level at which the map is displayed, ii) by specifying one or more categories of terms to be displayed from a desired level of hierarchy, iii) by choosing a subset of experimental conditions to be plotted. The selection of the summarization level is performed *via* a drop-down menu. Once the desired level is selected and the “Plot Map” button is clicked, the panel with the results is automatically updated, providing a new map where the rows correspond to the categories of the chosen level, grouped by their super classes in the hierarchy. The color of the cells in the new map is associated with the summarized value of all the enriched terms in the experimental condition column belonging to the category row.

The concept of level categories can be used to select subsets of rows of interest. This is done by selecting, for each represented level, the categories/terms of interest. The tool subsequently updates the map reporting only category/terms from the selected set, thus allowing a more compact view of the portion of interest of the map. Similarly, the user can specify a subset of experiments to be plotted.

Finally, the columns of the map can be reordered by grouping experimental conditions having similar enrichment profiles. This is accomplished by selecting a desired number of groups, a distance function among Jaccard, Euclidean and “Jaccard+Euclidean”, and a clustering linkage method between complete, single, and ward. In the “Clustering” sub-tab of “Plot Maps”, FunMappOne provides a visualization of the cluster dendrogram as well as the partitioning based on the number of desired clusters. This functionality can help in selecting the most appropriate number of clusters to be displayed. Finally, the current view of the map can be exported in various graphical formats.

We finally provide a comparison among FunMappOne features and those offered by a selection of currently available tools for functional annotation having close scope to FunMappOne. Table 1 shows the comparison of FunMappOne with the following gene functional analysis tools: DAVID [1], Enrichr [2], ToppGene [3], g:profiler [4], clusterProfiler [5], Goplot [6] and BACA [7]. As shown in Table 1, most of the other tools offer the possibility to analyze KEGG pathways, Reactome pathways and Gene Ontology, also with a graphic representation of the enrichment results. Only Goplot offers the possibility to map gene associated values to terms, while Enrichr and g:profiler are the only tools offering a web based graphical user interface. None of the other tools offer the possibility to summarize results and to cluster functional profiles from multiple experiments. To our knowledge, FunMappOne is the only tool providing all of these functionalities in a user friendly graphical interface.

**Table 1:** Comparison with existing tools

Feature/Tool	DAVID	Enrichr	ToppGene	g:profiler	clusterProfiler	Goplot	BACA	FunMappOne
KEGG pathways	$\checkmark $	$\checkmark $	$\checkmark $	$\checkmark $	$\checkmark $		$\checkmark $	$\checkmark $
Reactome pathways	$\checkmark $	$\checkmark $	$\checkmark $	$\checkmark $	$\checkmark $			$\checkmark $
Gene Ontology	$\checkmark $	$\checkmark $	$\checkmark $	$\checkmark $	$\checkmark $	$\checkmark $	$\checkmark $	$\checkmark $
Graphic representation		$\checkmark $	$\checkmark $		$\checkmark $	$\checkmark $	$\checkmark $	$\checkmark $
Graphic user interface	$\checkmark $	$\checkmark $		$\checkmark $				$\checkmark $
Hierachycal summarization								$\checkmark $
Multiple experiments					$\checkmark $		$\checkmark $	$\checkmark $
Term based clustering								$\checkmark $
Mapping values on terms						$\checkmark $		$\checkmark $

Different tools are reported on columns, desired features are reported on rows. Check-marks represent the presence of the feature in the tool

### Case study

We showcase the functionalities of FunMappOne on a transcriptome dataset of mouse hepatocytes exposed to 26 chemical compounds with different carcinogenic potential [11]. While Schaap et al. defined the similarity between the mechanism of action of a pair of chemicals at the level of individual genes, we tested the hypothesis that significant similarity patterns can be observed also at the functional annotation level. An excel file (Additional file 2) containing the originally described lists of the 30 most up-regulated and 30 most down-regulated genes in each compound-to-control comparison, along with the corresponding t-statistics, was uploaded to FunMappOne.

The annotation was performed by selecting the “KEGG” option and “gSCS” as multiple testing correction method with “0.05” as significance threshold (Additional file 3). For the plotting, the “median” function was chosen as summary statistics and colors were associated to the summarized modification direction of enriched terms by selecting the “sign” option (Additional file 3). Chemical exposures were finally ordered based on the “Jaccard” distance on the number of shared terms, and further clustered into 11 groups using hierarchical clustering and “complete” aggregation method.

Additional file 3 shows the KEGG enrichment map at the level 1 (Additional file 3A), level 2 (Additional file 3B), and at the individual pathway level 3 (Additional file 3C).

Our analysis confirmed many similarities originally described by Schaap and collaborators, such as the one between Wyeth-14643 (WY) and Clofibrate (CF), which in our analysis were grouped together with Tacrolimus (FK506) in cluster 11 (Additional file 3C). These chemicals modulate PPAR signalling pathway and fatty acid metabolism related genes, which we observed to be significantly enriched. Moreover, we identified a large cluster of compounds (cluster 6) characterized by no significantly enriched pathway, whose pairwise similarity of their mechanism of action were also described in the original report, but with a low significance [11].

Interestingly, enriched alteration of pathways related to steroid hormone biosynthesis and chemical carcinogenesis was observed in a group of known carcinogenic compounds clustered together (cluster 5). The visualizations produced at higher levels of the pathway hierarchy help the user to immediately observe that the chemicals in cluster 5 alter the genes in metabolic pathways and human diseases (Additional file 3A). When the visualization at level 2 is inspected, the notion that lipid metabolism and cancer pathways are enriched also easily emerges. This functionality of FunMappOne becomes very effective when analyzing richer functional annotations, such as gene ontology, where the number of enriched terms can be significantly higher (as shown in Additional file 4).

## Conclusion

We present FunMappOne, a web based standalone application that enables users to graphically inspect, navigate, and compare functional annotations in multiple experiments at different levels of abstraction. This tool facilitates the analyses of multiple experimental conditions through a simple user interface and dynamic graphical representations of the relevant functional categories. The FunMappOne software is open-source and distributed under the AGPL-3 license.

## Availability and requirements

**Project name:** FunMappOne

**Project home page:** https://github.com/Greco-Lab/FunMappOne

**Operating system(s):** Cross-platform

**Programming language:** R

**Other requirements:** Shiny

**License:** AGPL-3

**Any restrictions to use by non-academics:** For commercial use and modifications please contact the corresponding author.

## Additional files

Additional file 1 FunMappOne user manual. User manual for the FunMappOne tool. (DOCX 1940 kb)

Additional file 2 Excel file containing input data for the case study. The excel file is composed of one sheet for each exposure and a last sheet containing grouping information. Each exposure sheet is named with the exposure ID and contains two columns containing the list of selected genes and the associated t-statistics, respectively. The last sheet contains two columns: one reporting the list of exposure IDs and another the corresponding group. (XLSX 63 kb)

Additional file 3 Case study KEGG enrichment maps. KEGG enrichment maps showing modification direction after clustering analysis with 11 clusters. Panel A (top) shows enrichment results summarized at KEGG Level 1, panel B (middle) shows enrichment results summarized at KEGG Level 2, panel C (bottom) shows enrichment results summarized at KEGG Level 3 (pathways level). (PPTX 6869 kb)

Additional file 4 Level 1,2,3 Reactome and Gene Ontology (BP, CC, MF) maps for the proposed case study. Reactome maps have been produced by providing “Additional file 1” as input and choosing “Reactome” enrichment, annotation was performed using “Bonferroni” as multiple testing correction method with “0.001” as significance threshold. Three classes of Gene Ontology maps have been produced by providing “Additional file 1” as input and choosing “GO” and alternatively “BP”, “CC” or “MF” enrichment, annotation was performed using “Bonferroni” as multiple testing correction method with “0.001” as significance threshold. In both cases, for the plotting “median” was chosen as summary statistics and map colors were associated to the summarized each term modification direction by choosing the sign option. (PDF 3044 kb)

## Abbreviations

ARO: Aroclor 1254
BP: Biological processes
BPA: Bisphenol A
CA: Calyculin A
CC: Cellular components
CF: Clofibrate
CSA: Cyclosporin A
CSPT: Cisplatin
CT: Carbon tetrachloride
DEM: Diethyl maleate
DIDP: Diisodecyl phthalate
DM: D-mannitol
Enr.P: Term enrichment *p*-value
ETP: Etoposide
FK506: Tacrolimus
HCE: Heptachlor epoxide
HCH: *β-Hexachlorocyclohexane*
KEGG: Kyoto Encyclopedia of Genes and Genomes
LAC: Lead acetate
MEN: Menadione
MF: Molecular functions
MMC: Mitomycin C
MNU: N-Methyl-N-nitrosourea
MV: Gene modification value
OA: Okadaic acid
PB: Phenobarbital
SAR: Sodium arsenite
SS: Summary statistics
TBTO: Tributyltin oxide
TCE: 1,1,1,-Trichloroethane
TCDD: 2,3,7,8-Tetrachlorodibenzo-p-dioxin
TCPOBOP: 1,4-Bis[2-(3,5-dichloropyridyloxy)]benzene
WY: Wyeth-14643
