# Supplementary material for: FunMappOne: a tool to hierarchically organize and visually navigate functional gene annotations in multiple experiments
Source: BMC Bioinformatics. 2019 Feb 15;20:79. doi: 10.1186/s12859-019-2639-2 (PMC6376640; doi:10.1186/s12859-019-2639-2)
Supplement: Supplementary file 1 — FunMappOne user manual. User manual for the FunMappOne tool. (DOCX 1940 kb) [file 12859_2019_2639_MOESM1_ESM.docx]

FunMappOne Reference Manual

# Index

1. Input format
   1. Data format
   2. The experiment sheets
   3. The grouping sheet
2. Input tab
   1. Input gene lists
   2. Functional annotation parameters
   3. Display parameters
3. Plot Maps tab
   1. Plotting at different levels
   2. Cluster experiments
   3. Filter experiments
   4. Filter rows by level
   5. Download map

# Input format

- 1. **Data format**: Data should be provided in a single Excel file consisting of a separate sheet for each experimental condition.
  2. **The experiment sheets**: Each sheet should be named with a condition id and consist of two columns: the gene names (gene symbols, ensembl, or entrez) and gene modifications (T-statistic, fold change…).
  3. **The grouping sheet**: An additional sheet for grouping the conditions is compulsory. It must be the last sheet of the file and consist of two columns: first one with condition ids corresponding to the name of each sheet of the file (**d**.) and the second column for groupings. Groupings are formed by assigning the same number for each condition belonging to the same group (**f**.). If no predefined grouping will be provided, indicate only one number (e.g. fill the rows with number 1) for all conditions.


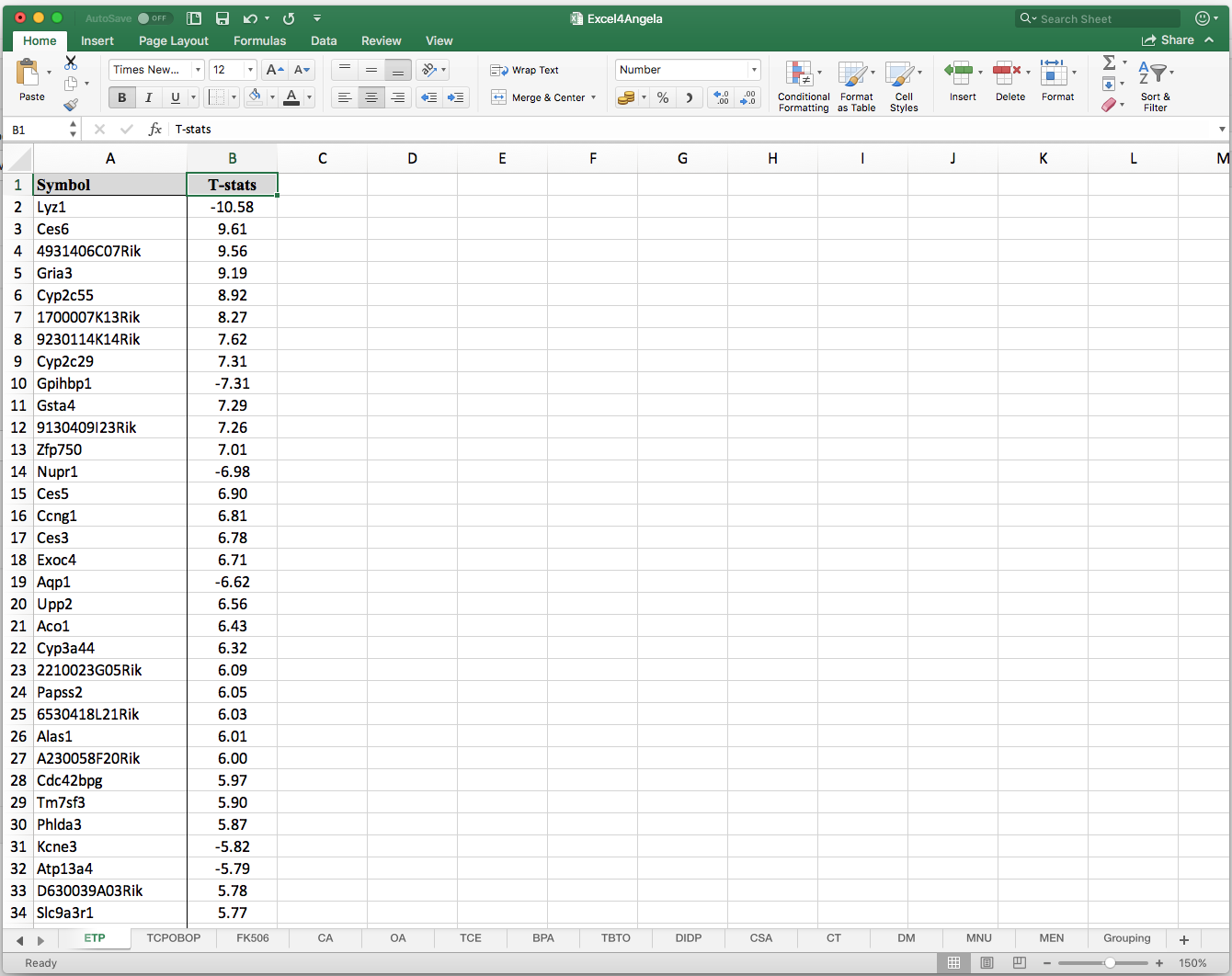


**b.**

**c.**

**a.**

**b.**

**d.**


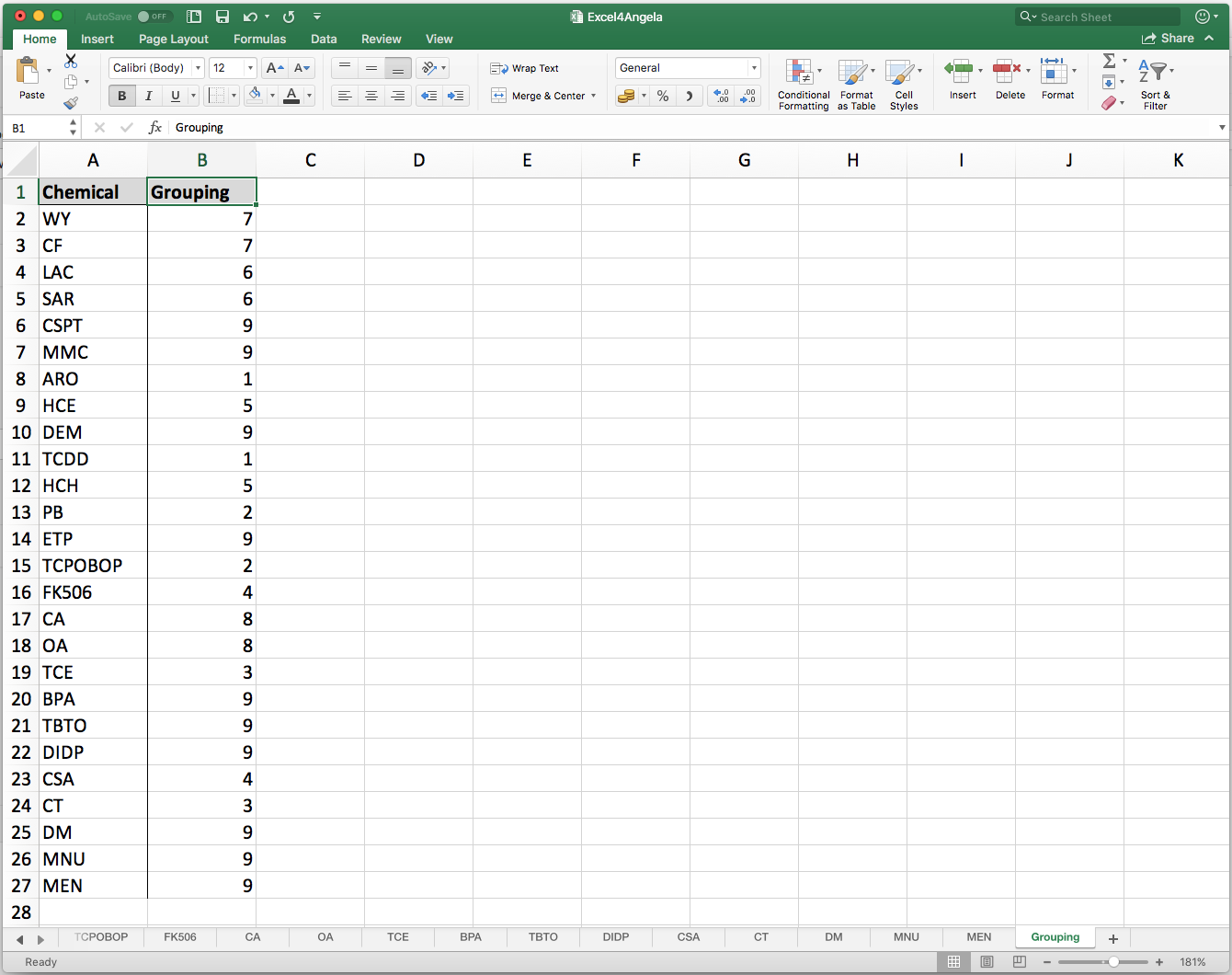


**f.**

# Input tab

- 1. **Input gene lists**: Select the organism, the type of gene names your list consists of and upload the Excel file in the format described above.
  2. **Functional annotation parameters**: In this section you can select the functional annotation between KEGG and Reactome pathways as well as GO and its aspects (Biological Processes, Cellular Components, Molecular Function). Select the value type for the annotation between the P-value of the enriched pathway, the gene modification provided in the input list, or the combination of the two. You can also adjust the threshold for the P-value.
  3. **Display parameters**: Select the display parameters. Choosing to plot based on the value will provide a colour scale of the chosen values while “sign” only indicates the direction of the modification.


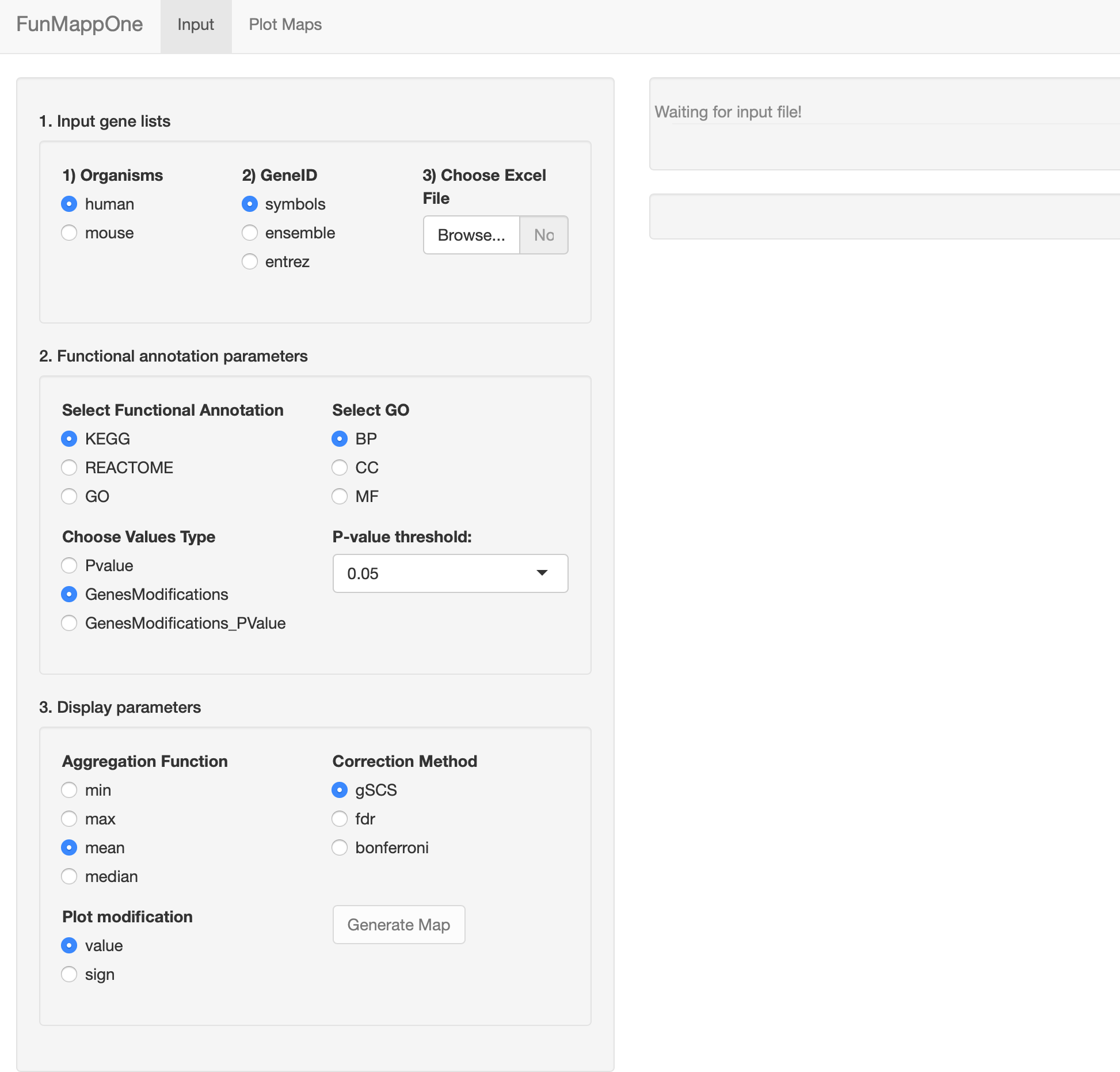


Upload a file

**c.**

**b.**

**a.**


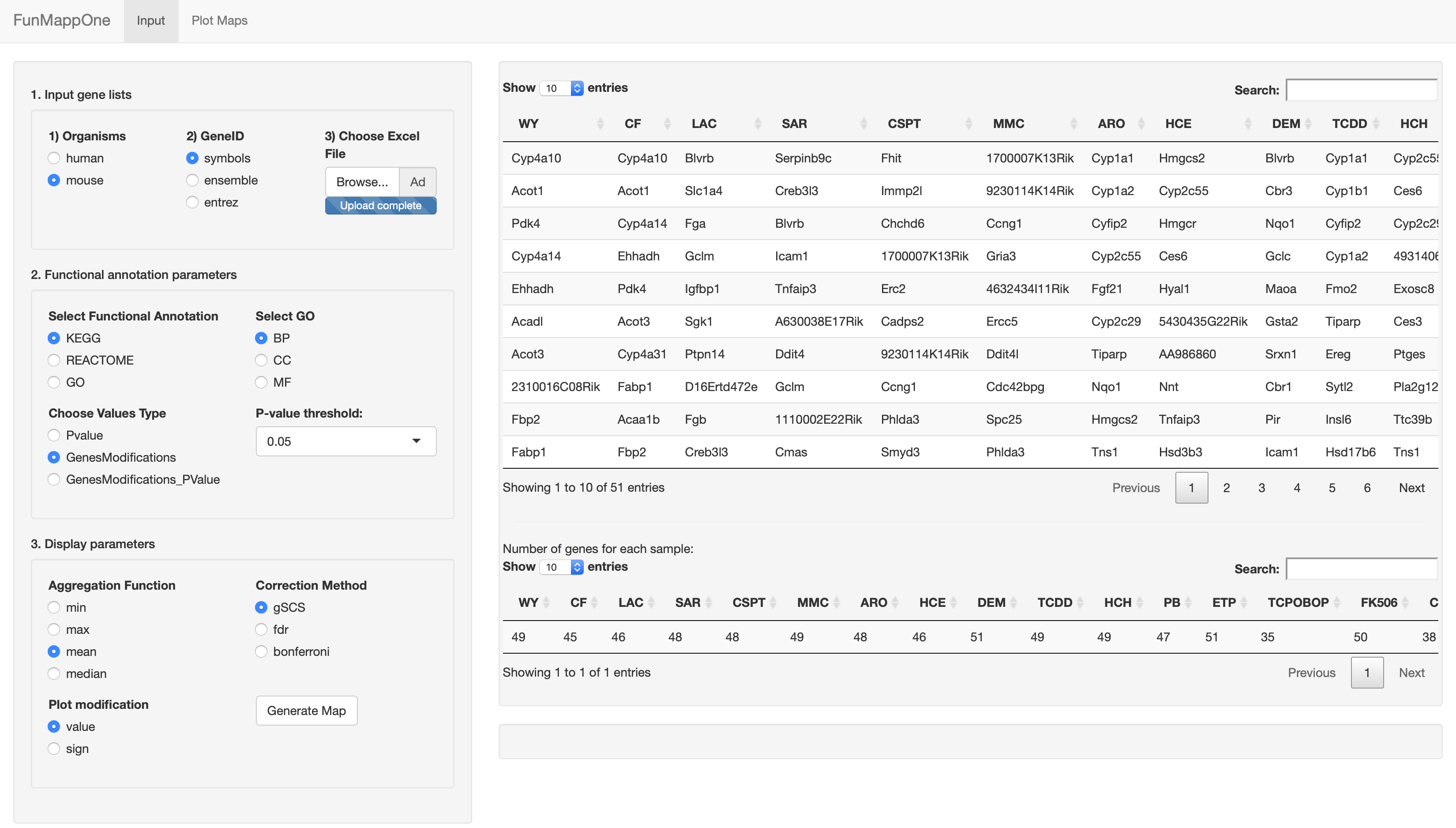


Generate Map will take you to the Plot Maps tab

The file has been successfully uploaded

# Plot Maps tab

- 1. **Plotting at different levels**: Enriched pathways and terms can be visualized in multiple levels of hierarchy.
  2. **Cluster experiments**: In Clustering Selection the experiments can be clustered into desired number of clusters with three different clustering methods and distances. Click “Cluster Samples” to activate clustering.
  3. **Filter experiments**: Experimental conditions (condition ids) can be filtered and only selected experiments will be mapped. Remove “All” from the selections to only plot the selected experiments.
  4. **Filter rows by level**: Pathways and processes can be filtered based on the level to only visualize the selected rows. Remove “All” from the selections to only plot the selected experiments.
  5. **Download map**: Map view can be downloaded as in pdf format by clicking “Download”.


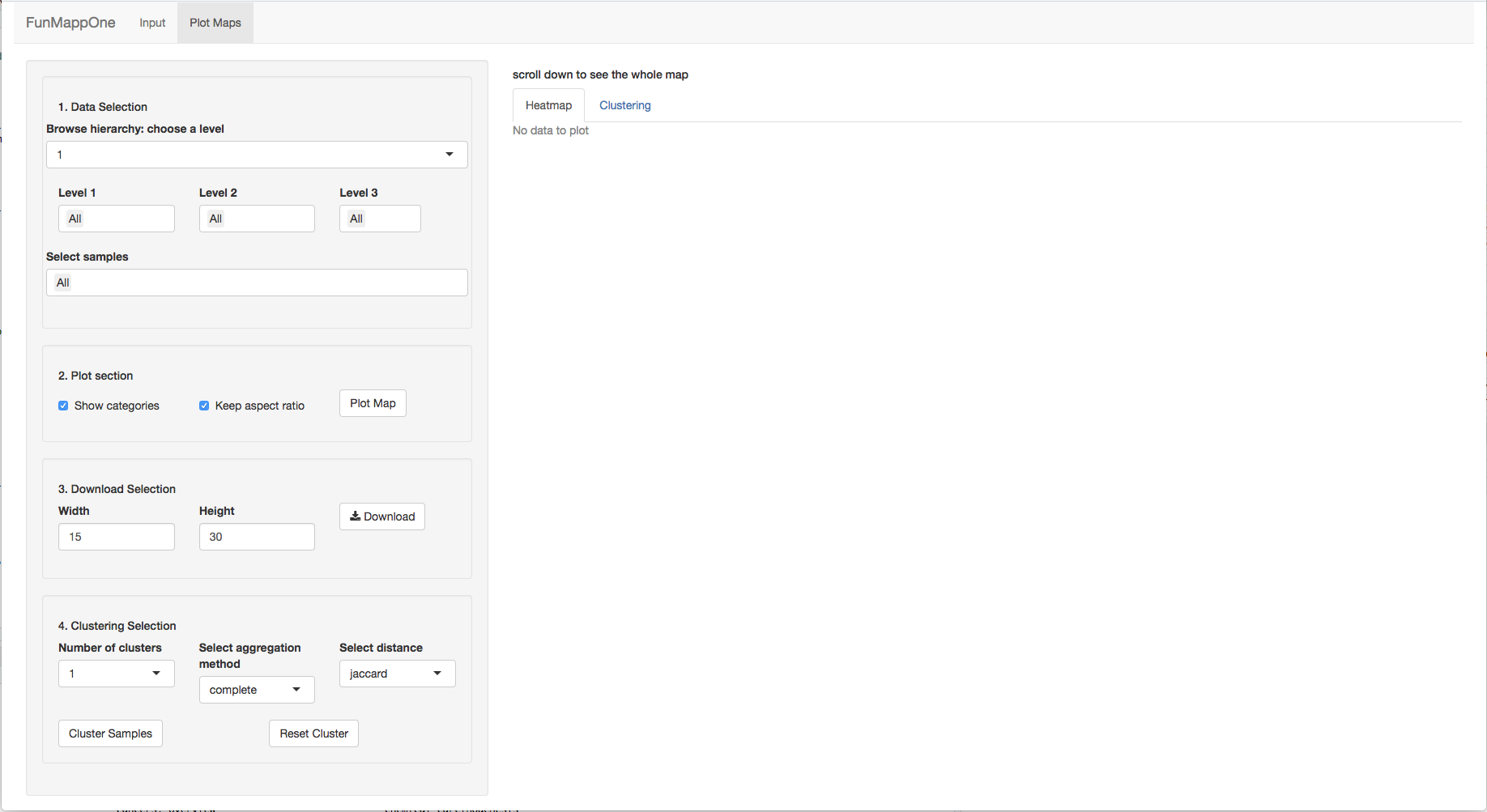


Click “Plot Map” to plot the map every time selections have been updated

**a.**

**d.**

**c.**

**e.**

**b.**


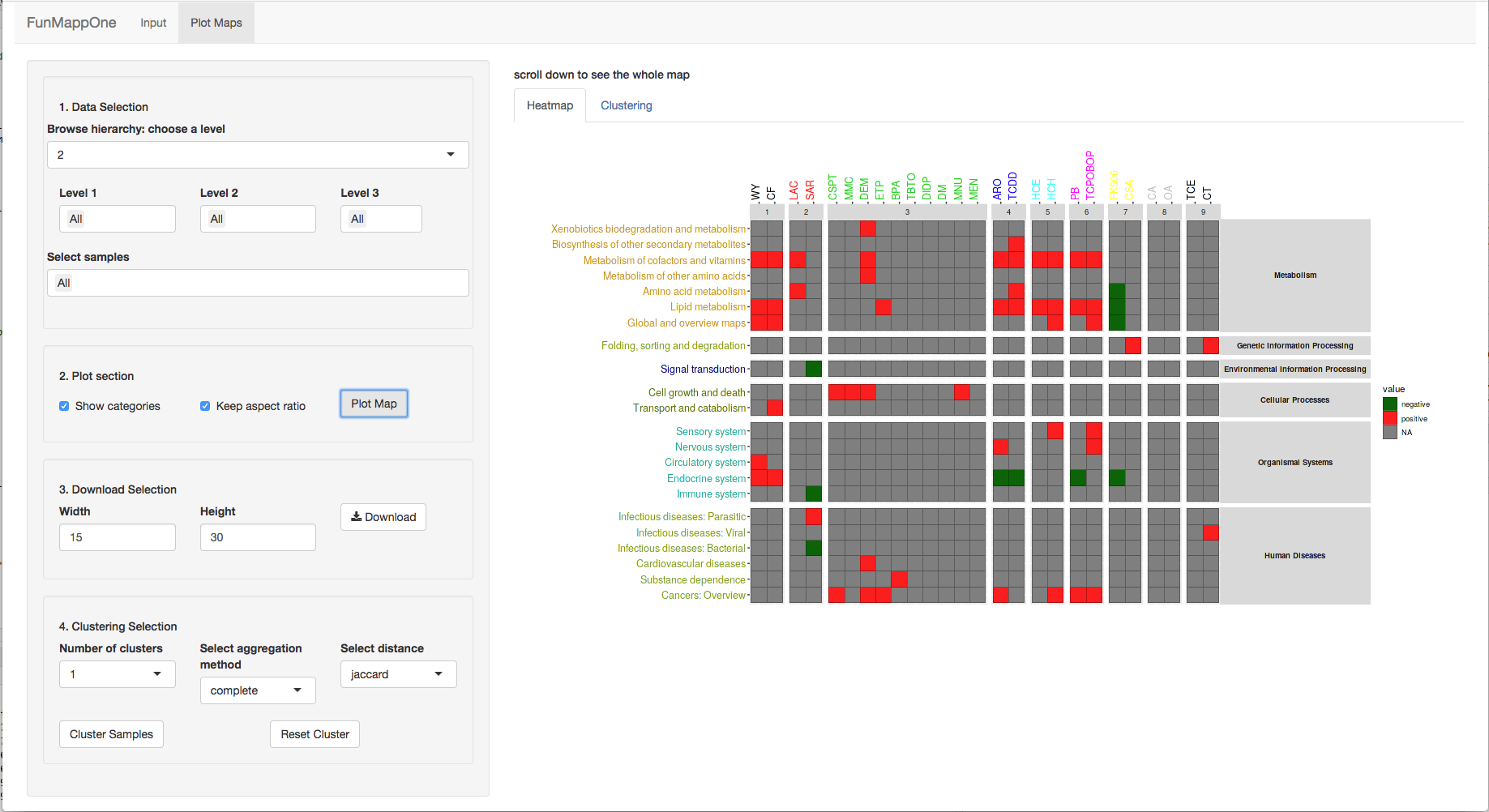


The data is now mapped on level 2. Before clustering the samples, the samples are grouped by the predefined groupings provided in the Excel file.

Unticking “Show categories” will remove the categories shown on the right side of the map

Condition ids


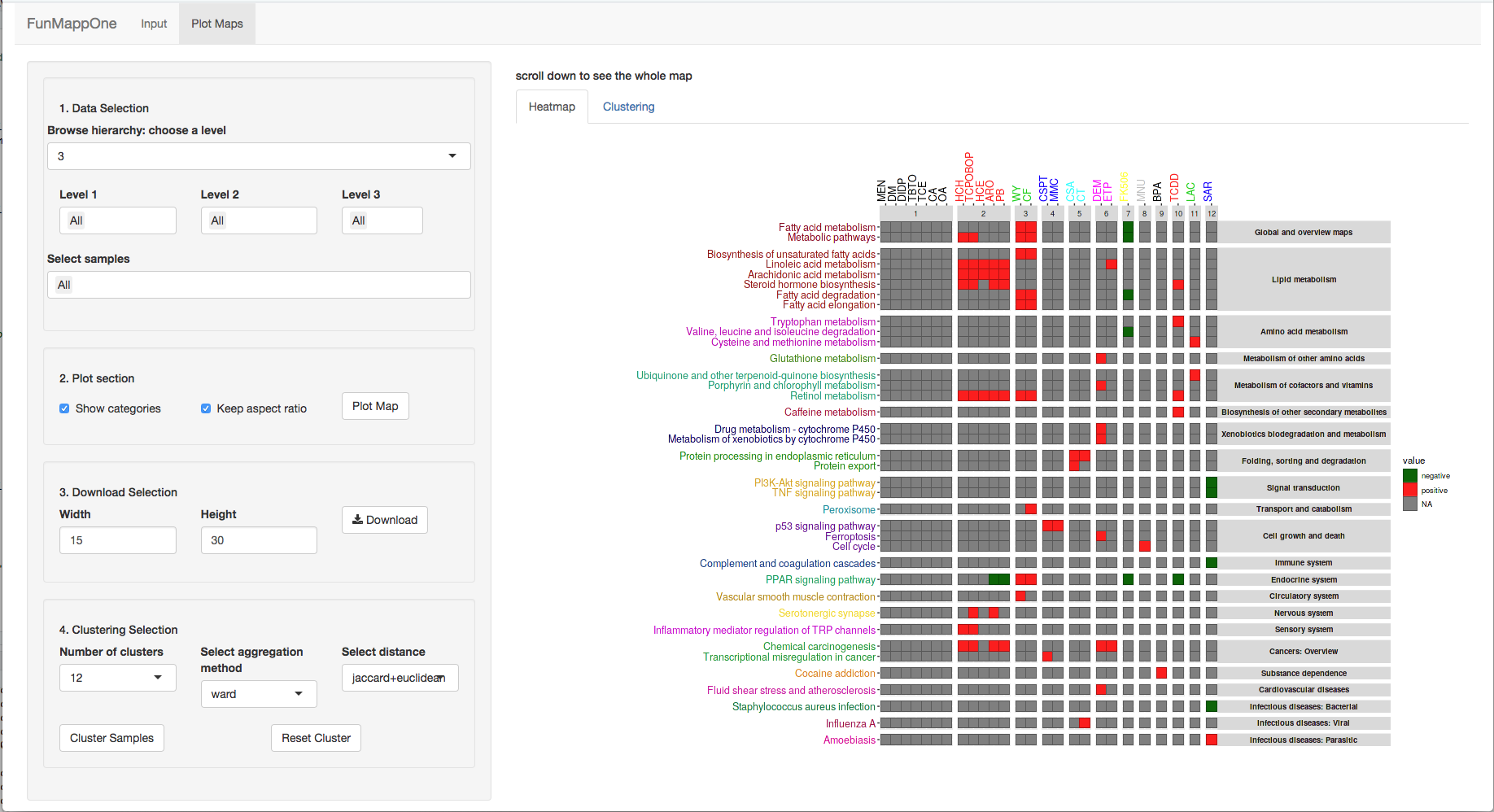


The data is now mapped on level 3 and clustered into 12 clusters.

**b.**


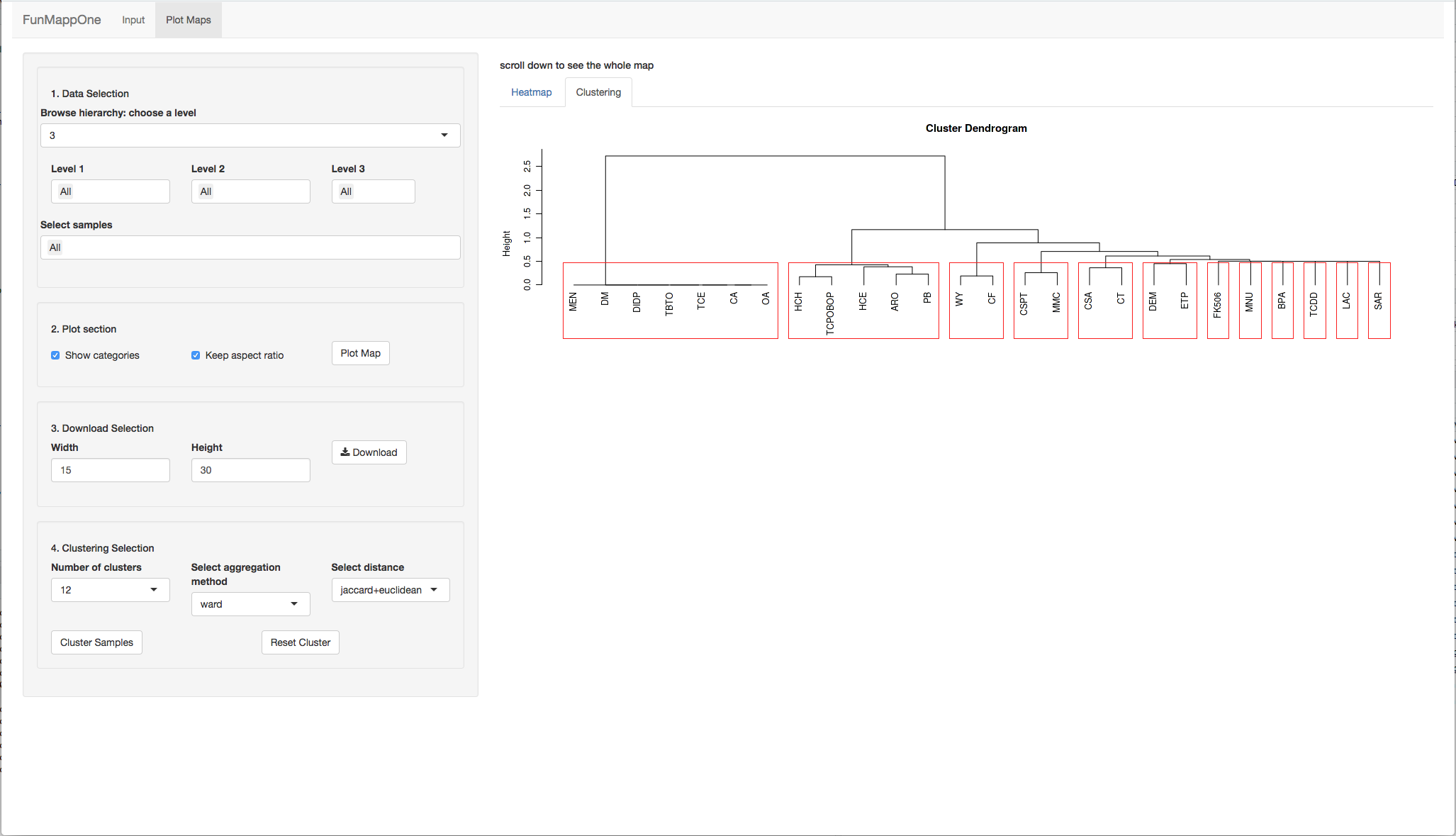


Cluster dendrogram can be visualized by clicking the Clustering tab
