# Supplementary figures and images for: FunMappOne: a tool to hierarchically organize and visually navigate functional gene annotations in multiple experiments
Source: BMC Bioinformatics. 2019 Feb 15;20:79. doi: 10.1186/s12859-019-2639-2 (PMC6376640; doi:10.1186/s12859-019-2639-2)

## Slide 1
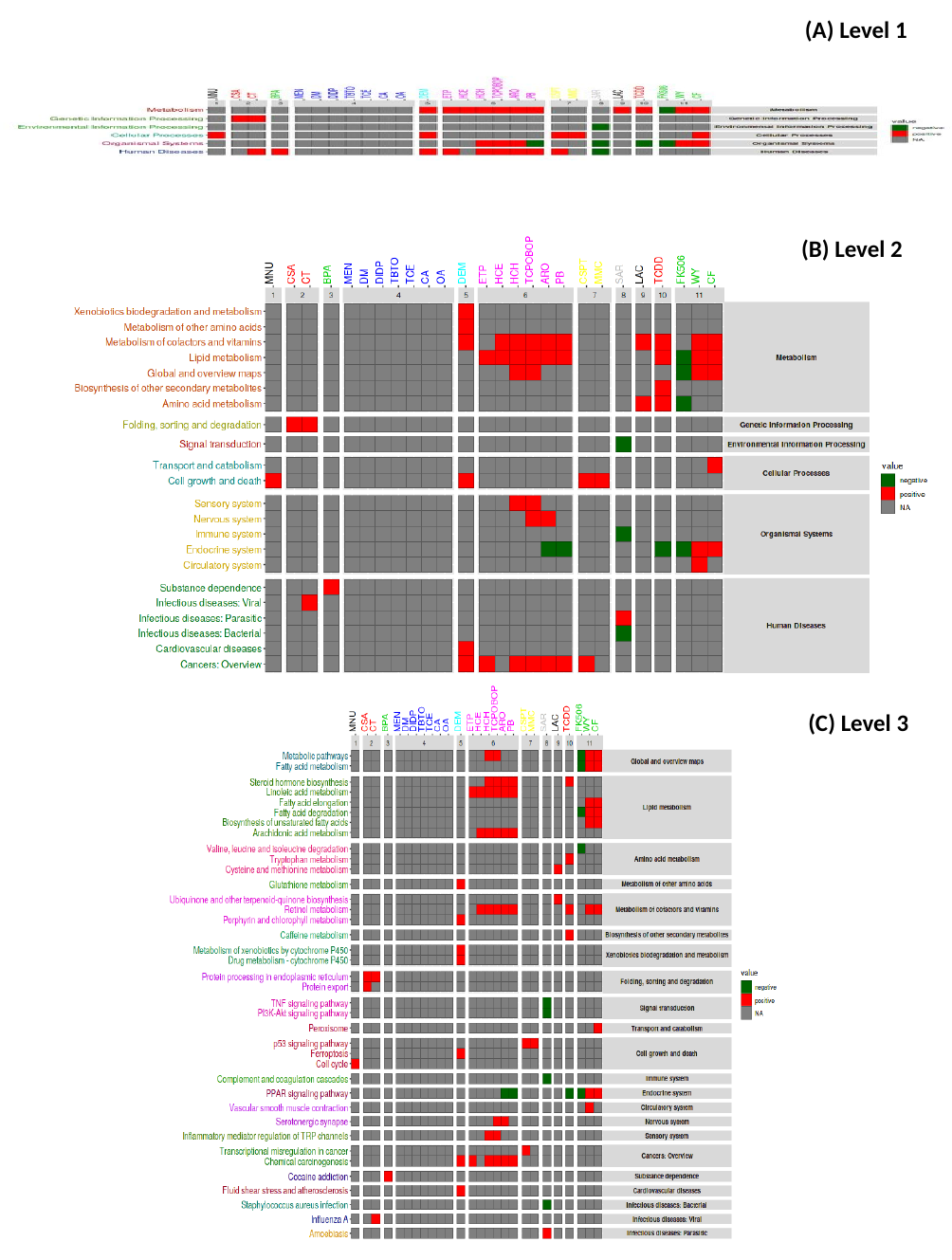

(A) Level 1
(B) Level 2
(C) Level 3

Supplement: Supplementary file 3 — Case study KEGG enrichment maps. KEGG enrichment maps showing modification direction after clustering analysis with 11 clusters. Panel A (top) shows enrichment results summarized at KEGG Level 1, panel B (middle) shows enrichment results summarized at KEGG Level 2, panel C (bottom) shows enrichment results summarized at KEGG Level 3 (pathways level). (PPTX 6869 kb) [file 12859_2019_2639_MOESM3_ESM.pptx]
