# Supplementary material for: FunMappOne: a tool to hierarchically organize and visually navigate functional gene annotations in multiple experiments
Source: BMC Bioinformatics. 2019 Feb 15;20:79. doi: 10.1186/s12859-019-2639-2 (PMC6376640; doi:10.1186/s12859-019-2639-2)

REACTOME Level 3

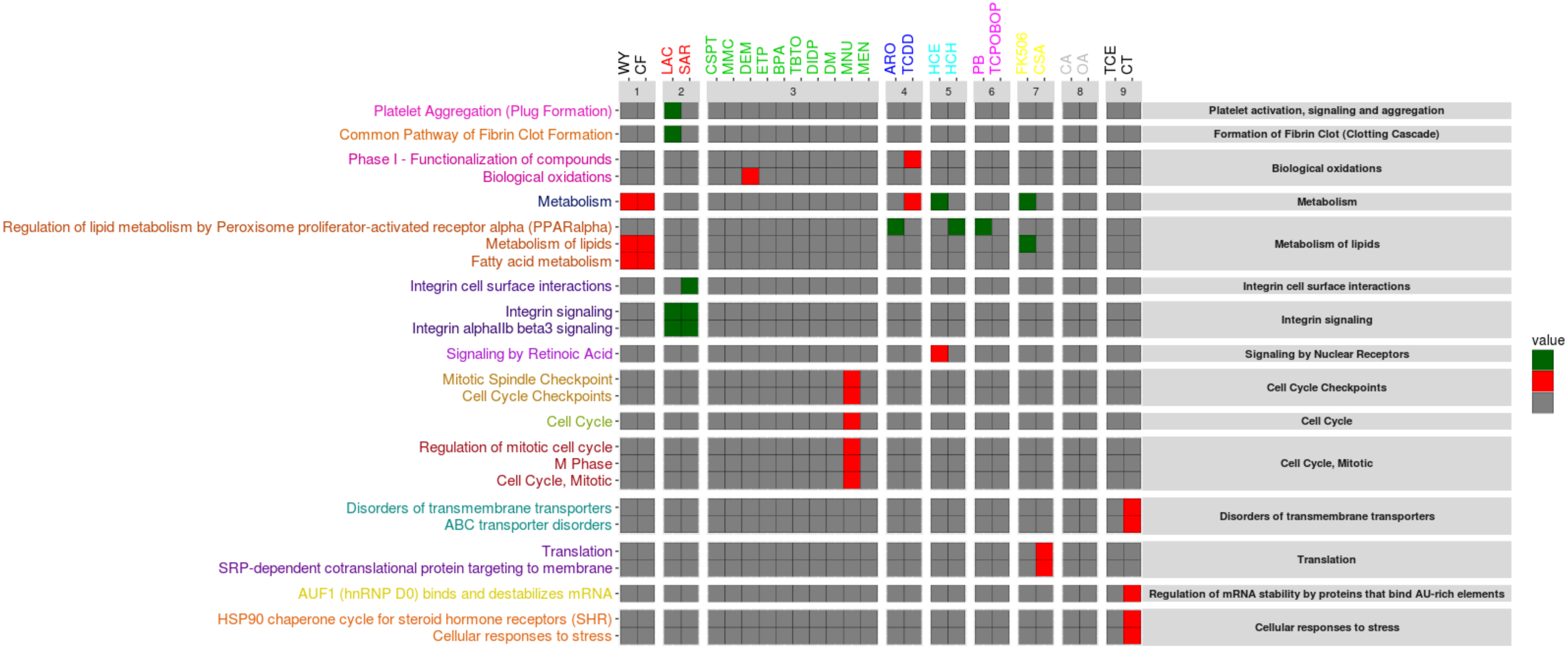

REACTOME   Level 2

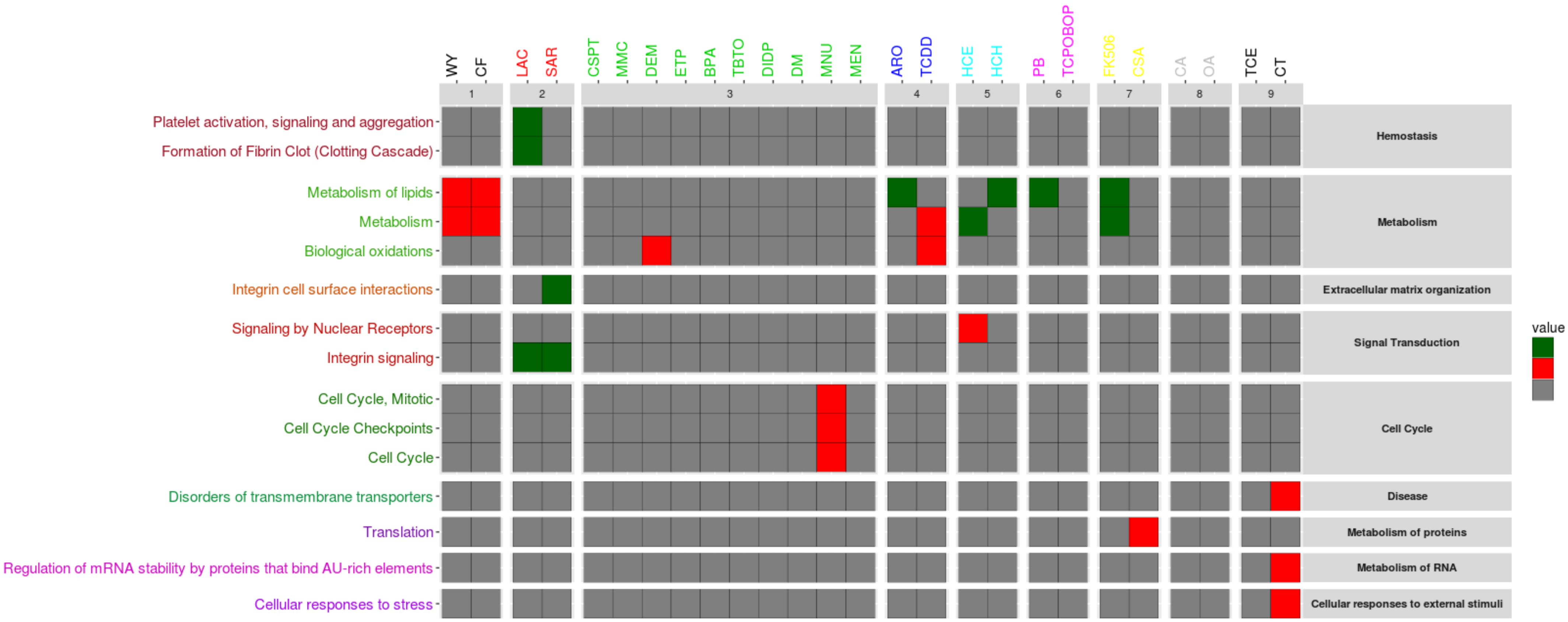

REACTOME Level 1

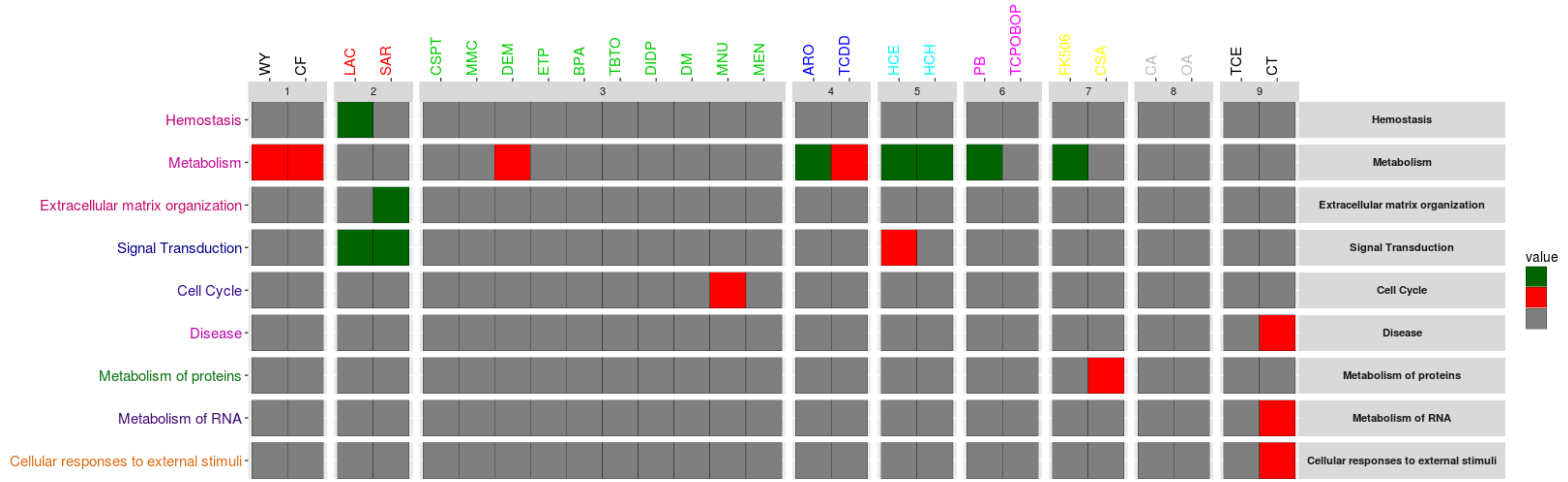

Gene Ontology-Biological Processes Level 3

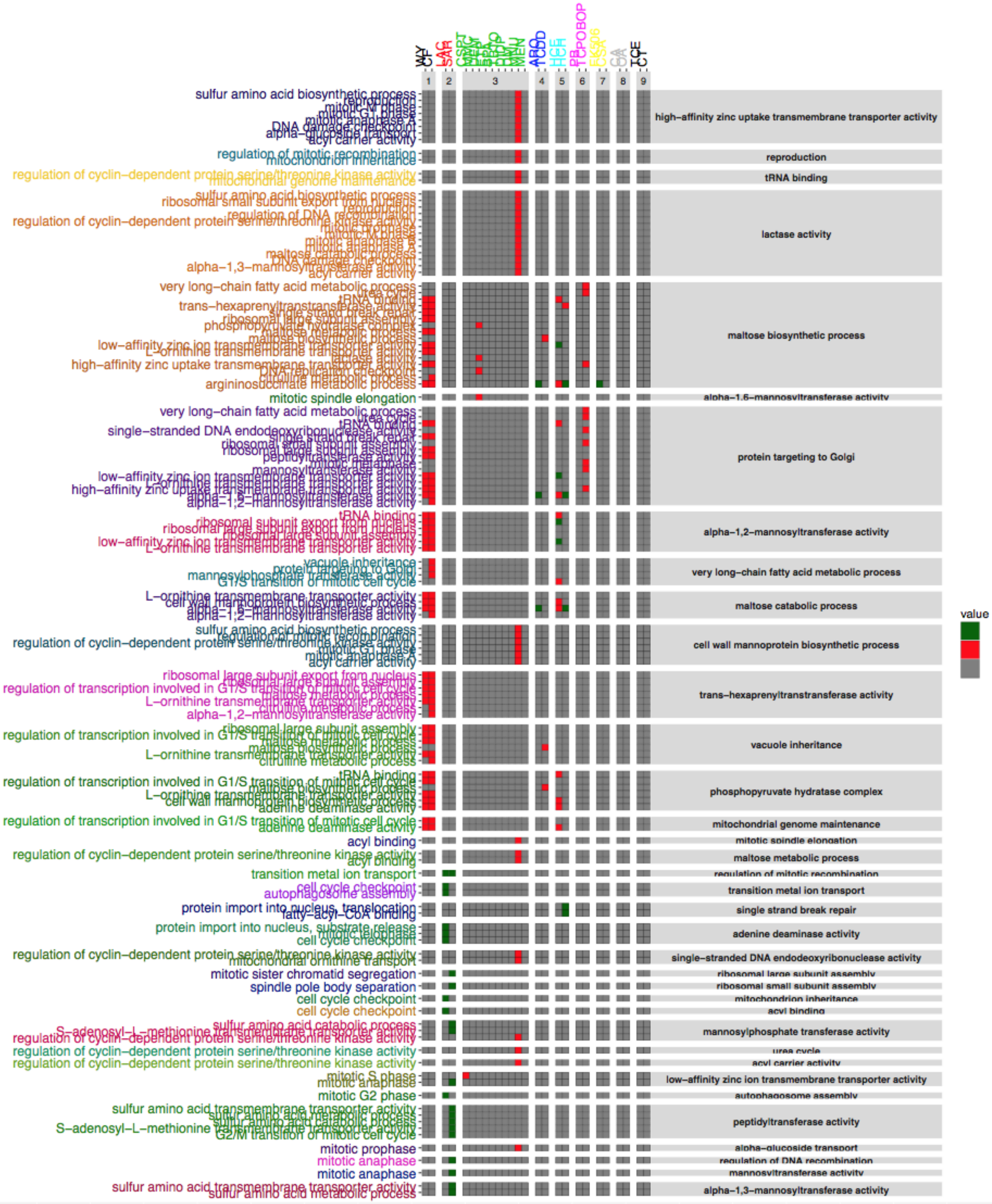

Gene Ontology-Biological Processes Level 2

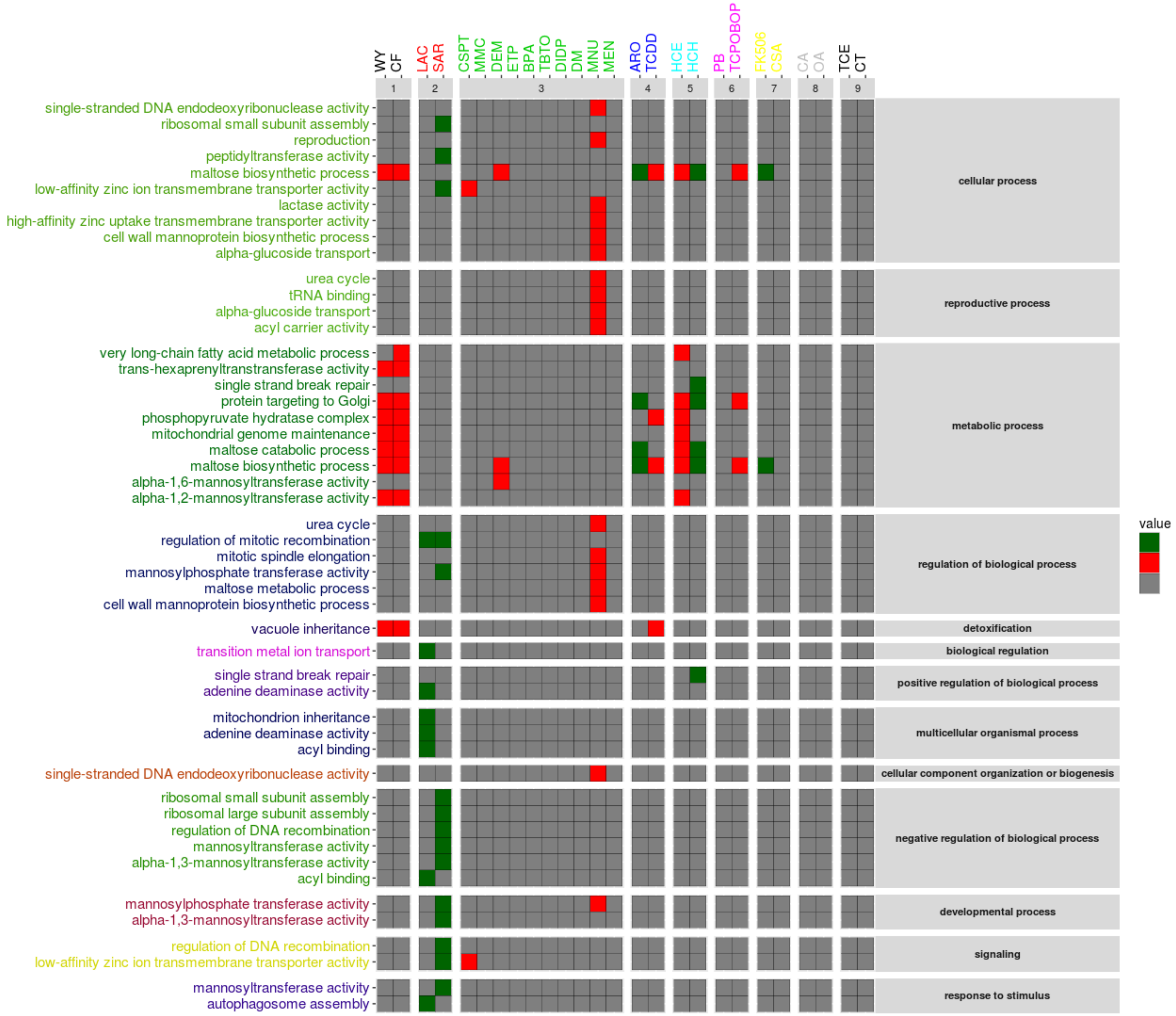

Gene Ontology-Biological Processes Level 1

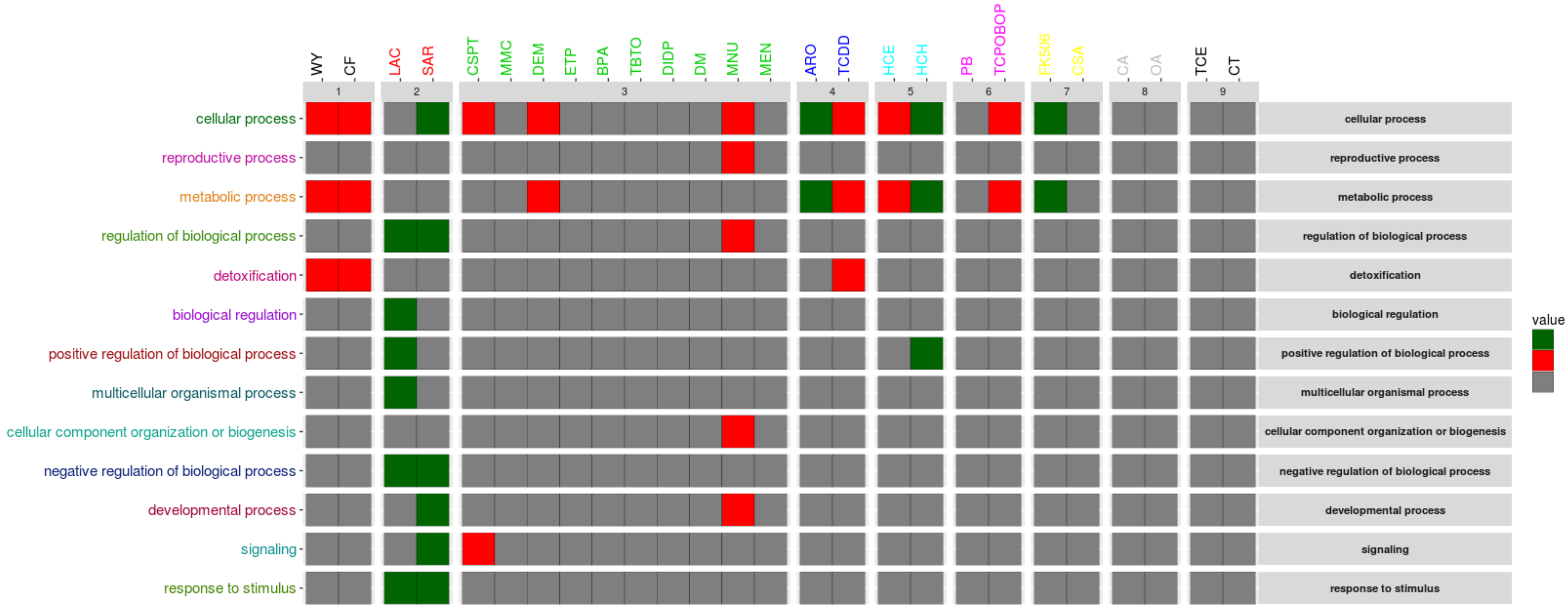

Gene Ontology-Cellular Components Level 3

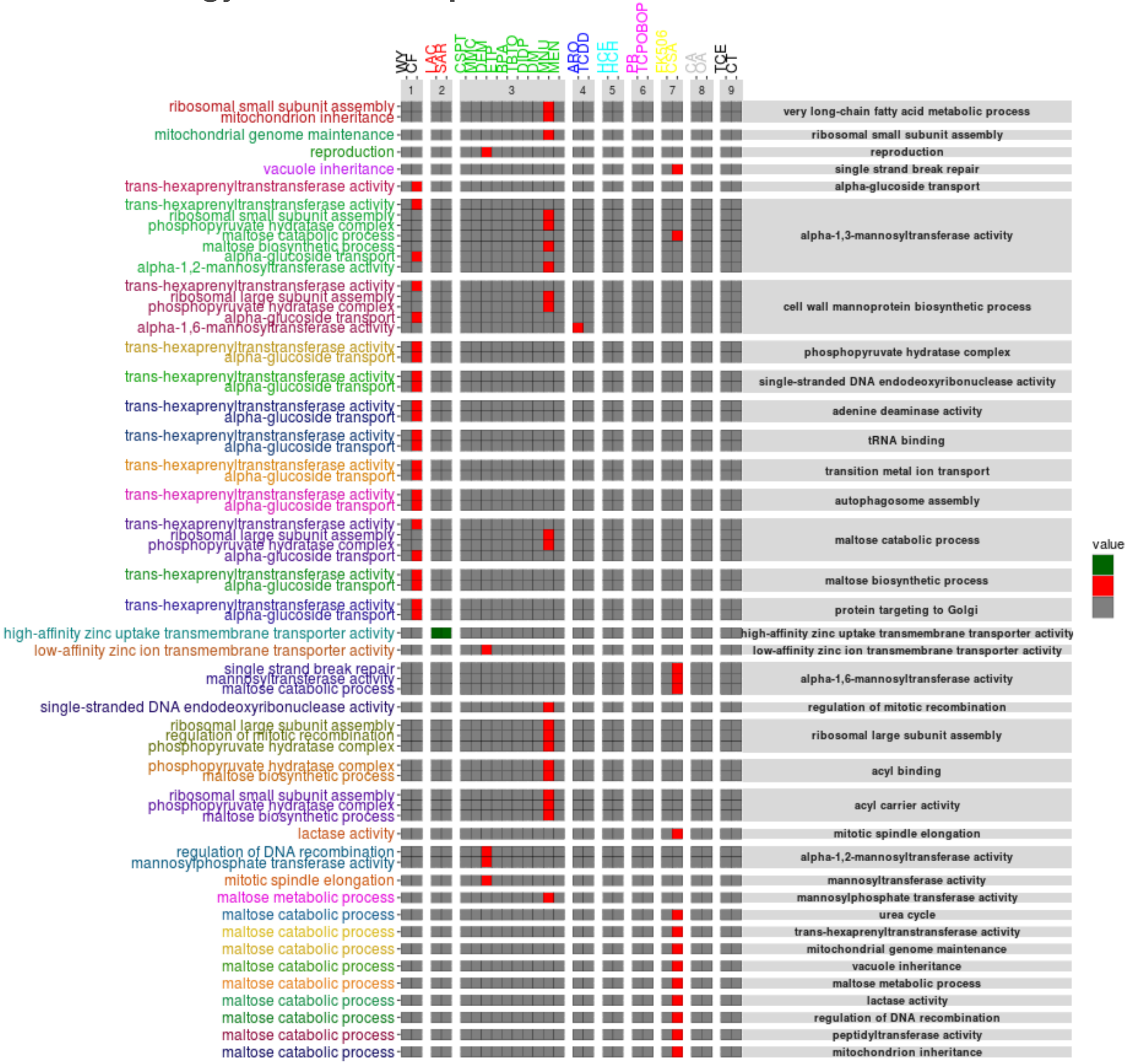

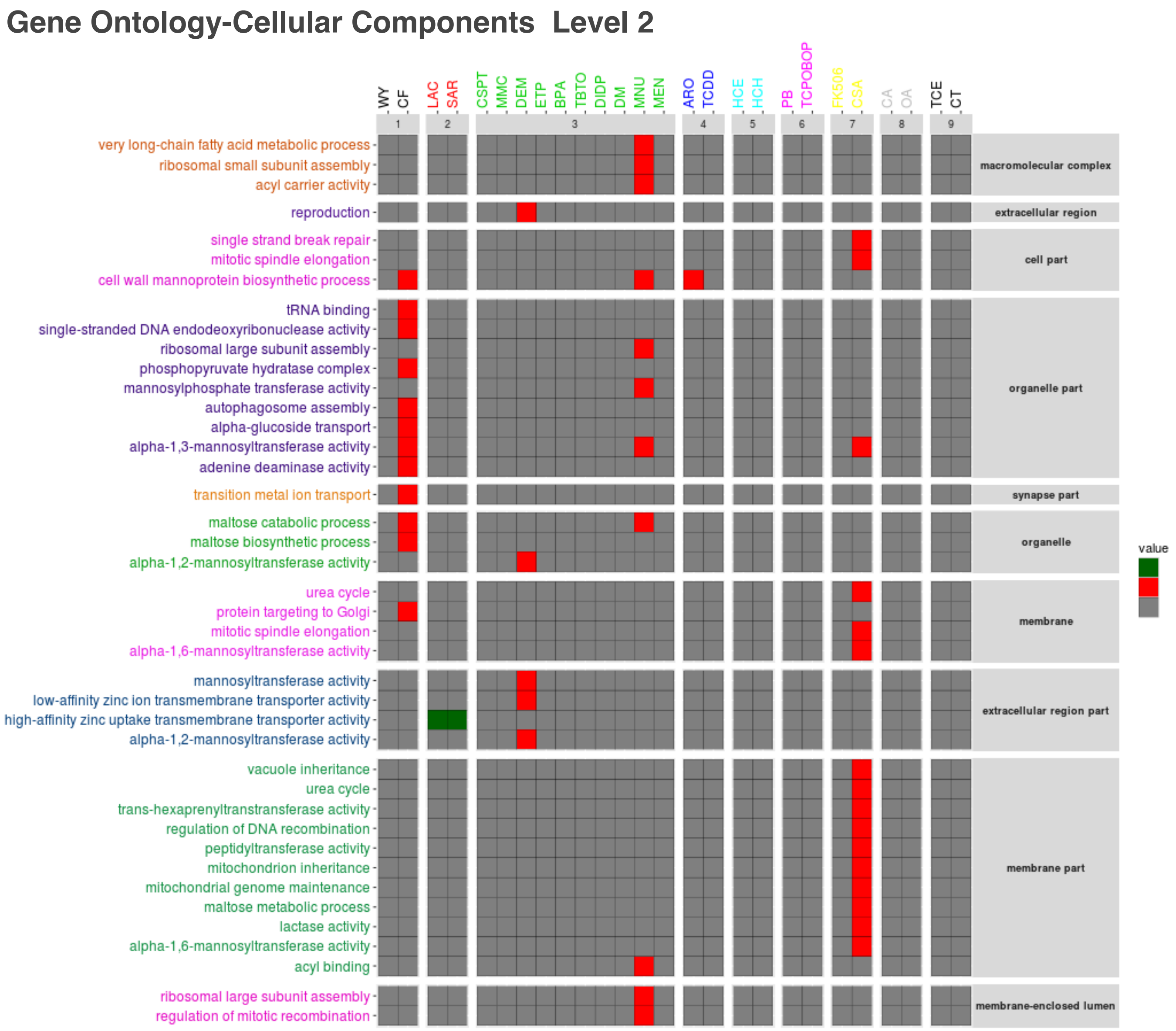

Gene Ontology-Cellular Components Level 2
WY
CF
LAC
SAR
CSPT
MMC
DEM
ETP
BPA
TBTO
DIDP
DM
MNU
MEN
ARO
TCDD
HCE
HCH
PB
TCPOBOP
FK506
CSA
CA
OA
TCE
CT
1
2
3
4
5
6
7
8
9
very long-chain fatty acid metabolic process
ribosomal small subunit assembly
acyl carrier activity
reproduction
single strand break repair
mitotic spindle elongation
cell wall mannoprotein biosynthetic process
tRNA binding
single-stranded DNA endodeoxyribonuclease activity
ribosomal large subunit assembly
phosphopyruvate hydratase complex
mannosylphosphate transferase activity
autophagosome assembly
alpha-glucoside transport
alpha-1,3-mannosyltransferase activity
adenine deaminase activity
transition metal ion transport
maltose catabolic process
maltose biosynthetic process
alpha-1,2-mannosyltransferase activity
urea cycle
protein targeting to Golgi
mitotic spindle elongation
alpha-1,6-mannosyltransferase activity
mannosyltransferase activity
low-affinity zinc ion transmembrane transporter activity
high-affinity zinc uptake transmembrane transporter activity
alpha-1,2-mannosyltransferase activity
vacuole inheritance
urea cycle
trans-hexaprenyltranstransferase activity
regulation of DNA recombination
peptidyltransferase activity
mitochondrion inheritance
mitochondrial genome maintenance
maltose metabolic process
lactase activity
alpha-1,6-mannosyltransferase activity
acyl binding
ribosomal large subunit assembly
regulation of mitotic recombination
macromolecular complex
extracellular region
cell part
organelle part
synapse part
organelle
membrane
extracellular region part
membrane part
membrane-enclosed lumen
value

Gene Ontology-Cellular Components Level 1

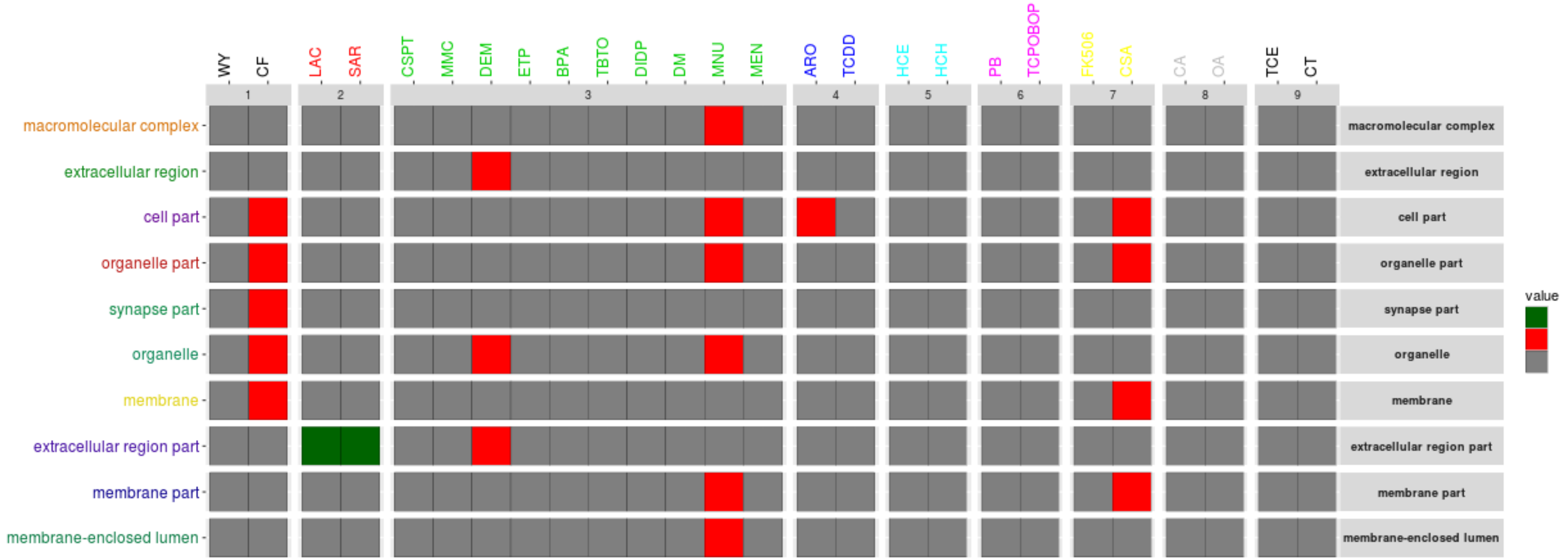

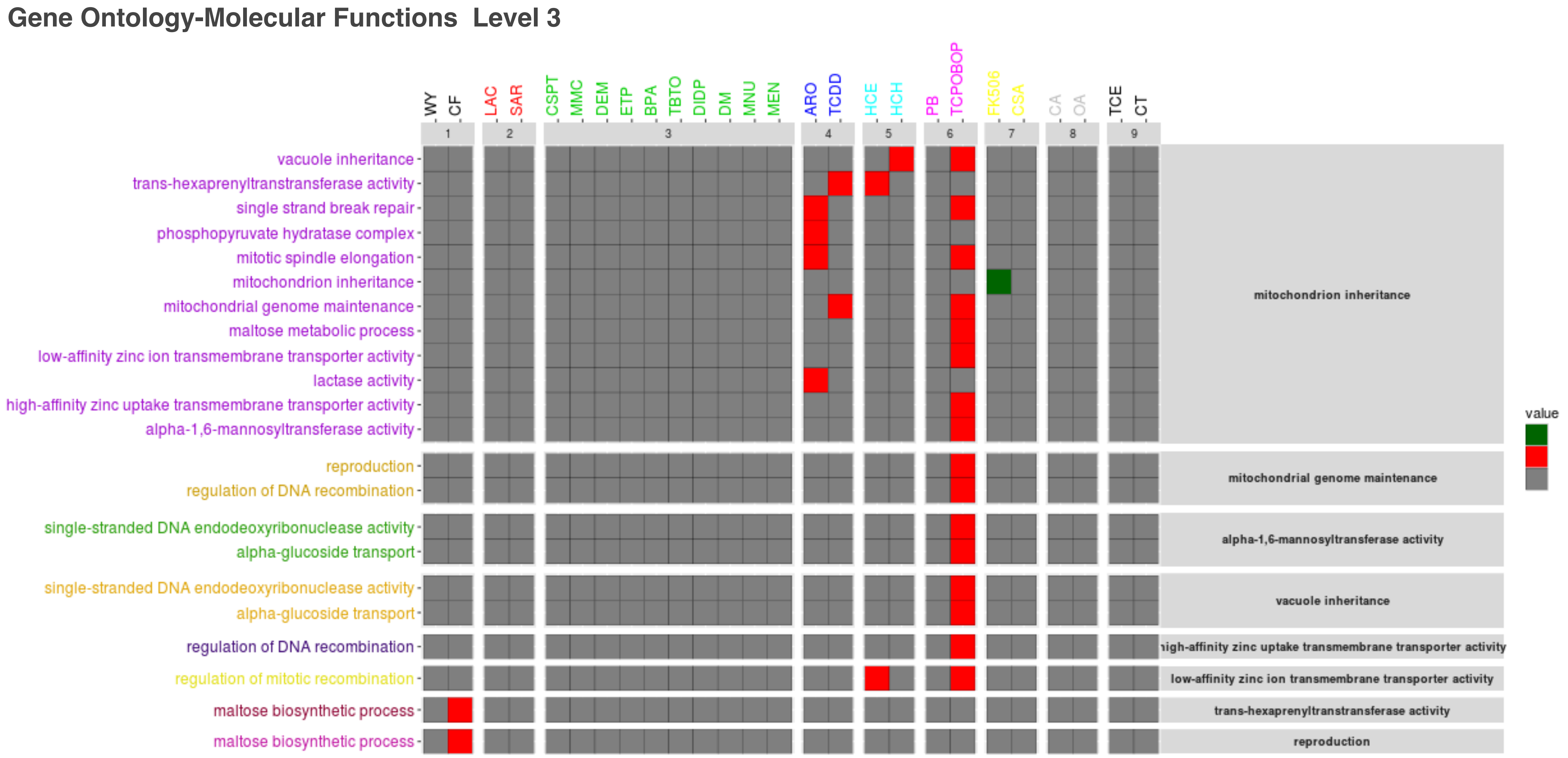

Gene Ontology-Molecular Functions Level 3
WY
CF
LAC
SAR
CSPT
MMC
DEM
ETP
BPA
TBTO
DIDP
DM
MNU
MEN
ARO
TCDD
HCE
HCH
PB
TCPOBOP
FK506
CSA
CA
OA
TCE
CT
1
2
3
4
5
6
7
8
9
vacuole inheritance
trans-hexaprenyltranstransferase activity
single strand break repair
phosphopyruvate hydratase complex
mitotic spindle elongation
mitochondrion inheritance
mitochondrial genome maintenance
maltose metabolic process
low-affinity zinc ion transmembrane transporter activity
lactase activity
high-affinity zinc uptake transmembrane transporter activity
alpha-1,6-mannosyltransferase activity
reproduction
regulation of DNA recombination
single-stranded DNA endodeoxyribonuclease activity
alpha-glucoside transport
single-stranded DNA endodeoxyribonuclease activity
alpha-glucoside transport
regulation of DNA recombination
regulation of mitotic recombination
maltose biosynthetic process
maltose biosynthetic process
mitochondrion inheritance
mitochondrial genome maintenance
alpha-1,6-mannosyltransferase activity
vacuole inheritance
high-affinity zinc uptake transmembrane transporter activity
low-affinity zinc ion transmembrane transporter activity
trans-hexaprenyltranstransferase activity
reproduction
value

Gene Ontology-Molecular Functions Level 2

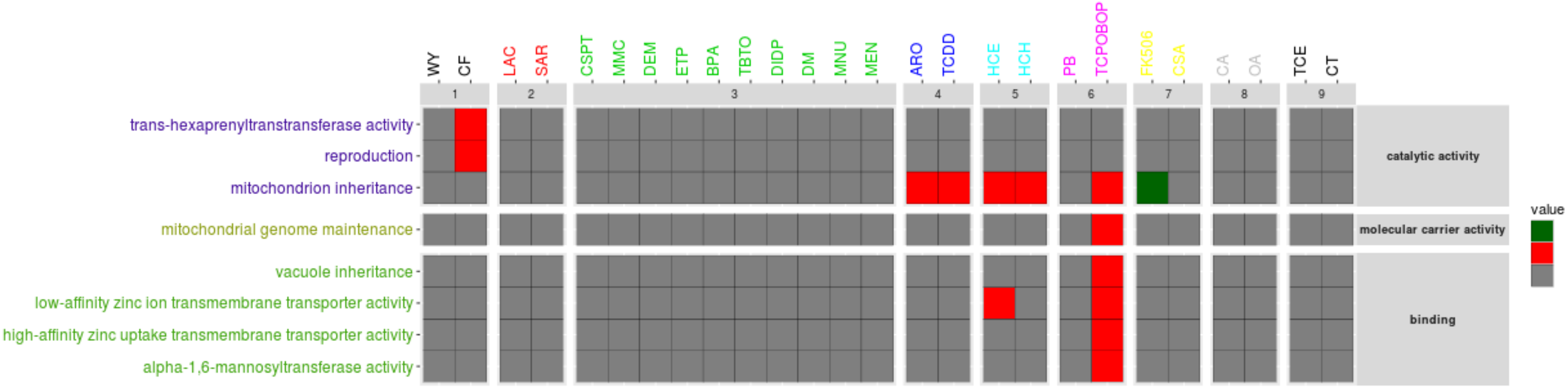

Gene Ontology-Molecular Functions Level1

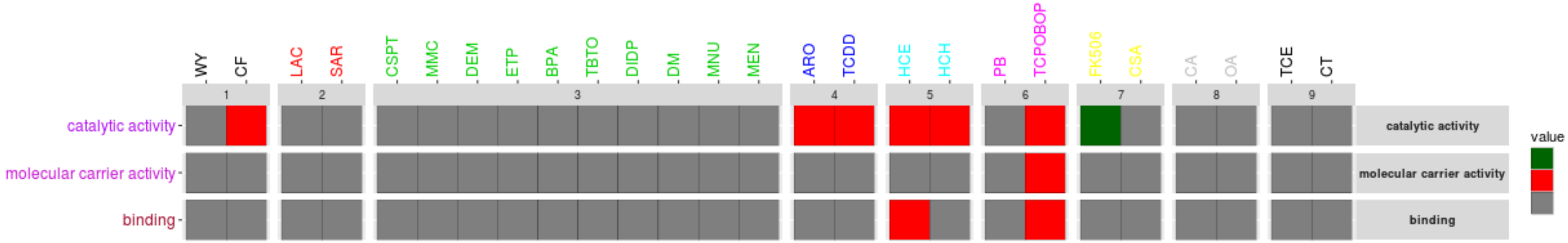

Supplement: Supplementary file 4 — Level 1,2,3 Reactome and Gene Ontology (BP, CC, MF) maps for the proposed case study. Reactome maps have been produced by providing “Additional file 1” as input and choosing “Reactome” enrichment, annotation was performed using “Bonferroni” as multiple testing correction method with “0.001” as significance threshold. Three classes of Gene Ontology maps have been produced by providing “Additional file 1” as input and choosing “GO” and alternatively “BP”, “CC” or “MF” enrichment, annotation was performed using “Bonferroni” as multiple testing correction method with “0.001” as significance threshold. In both cases, for the plotting “median” was chosen as summary statistics and map colors were associated to the summarized each term modification direction by choosing the sign option. (PDF 3044 kb) [file 12859_2019_2639_MOESM4_ESM.pdf]
